# Imaging of Small Animal Peripheral Artery Disease Models: Recent Advancements and Translational Potential

**DOI:** 10.3390/ijms160511131

**Published:** 2015-05-18

**Authors:** Jenny B. Lin, Evan H. Phillips, Ti’Air E. Riggins, Gurneet S. Sangha, Sreyashi Chakraborty, Janice Y. Lee, Roy J. Lycke, Clarissa L. Hernandez, Arvin H. Soepriatna, Bradford R. H. Thorne, Alexa A. Yrineo, Craig J. Goergen

**Affiliations:** 1 Weldon School of Biomedical Engineering, Purdue University, 206 S. Martin Jischke Drive, Room 3025, West Lafayette, IN 47907, USA; E-Mails: lin371@purdue.edu (J.B.L.); phill128@purdue.edu (E.H.P.); triggins@purdue.edu (T.E.R.); gsangha@purdue.edu (G.S.S.); rlycke@purdue.edu (R.J.L.); herna171@purdue.edu (C.L.H); asoepria@purdue.edu (A.H.S.); ayrineo@purdue.edu (A.A.Y.); 2 School of Mechanical Engineering, Purdue University, West Lafayette, IN 47907, USA; E-Mail: chakrab3@purdue.edu; 3 Psychological Sciences, Purdue University, West Lafayette, IN 47907, USA; E-Mail: jyxlee@purdue.edu; 4 School of Sciences, Neuroscience, Purdue University, West Lafayette, IN 47907, USA; E-Mail: thorneb@purdue.edu

**Keywords:** peripheral artery disease, aneurysm, atherosclerosis, ischemia, stroke, small animal model, ultrasound, magnetic resonance, computed tomography, optical imaging

## Abstract

Peripheral artery disease (PAD) is a broad disorder encompassing multiple forms of arterial disease outside of the heart. As such, PAD development is a multifactorial process with a variety of manifestations. For example, aneurysms are pathological expansions of an artery that can lead to rupture, while ischemic atherosclerosis reduces blood flow, increasing the risk of claudication, poor wound healing, limb amputation, and stroke. Current PAD treatment is often ineffective or associated with serious risks, largely because these disorders are commonly undiagnosed or misdiagnosed. Active areas of research are focused on detecting and characterizing deleterious arterial changes at early stages using non-invasive imaging strategies, such as ultrasound, as well as emerging technologies like photoacoustic imaging. Earlier disease detection and characterization could improve interventional strategies, leading to better prognosis in PAD patients. While rodents are being used to investigate PAD pathophysiology, imaging of these animal models has been underutilized. This review focuses on structural and molecular information and disease progression revealed by recent imaging efforts of aortic, cerebral, and peripheral vascular disease models in mice, rats, and rabbits. Effective translation to humans involves better understanding of underlying PAD pathophysiology to develop novel therapeutics and apply non-invasive imaging techniques in the clinic.

## 1. Introduction

**Figure 1:**
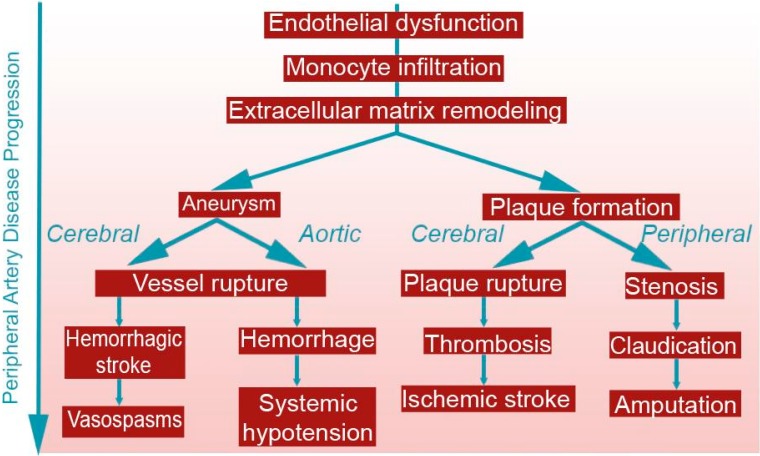
Disease Progression of peripheral artery disease (PAD). Many of the underlying factors of aneurysm and atherosclerosis progression are similar, regardless of arterial differences in the brain, abdomen, and limbs. The left branch represents aneurysm progression, while the right branch represents atherosclerosis progression.

Peripheral artery disease (PAD) encompasses a broad range of vascular pathologies in the extracoronary circulation, extending from the cerebral vasculature to lower limb arteries, and includes atherosclerosis, aneurysms, and arteriovenous malformations (Figure 1). Despite much technological advancement, a significant amount of mortality and morbidity from PAD exists, even before diagnosis or treatment thresholds are met [1,2]. Imaging techniques studying PAD progression have the capability to improve this status quo, facilitating earlier detection, guiding patient management decisions, and possibly enabling disease prevention.

The aim of this review is to analyze recent advancements in the field of PAD pathophysiology using various imaging modalities in small animal models. We will focus on pathophysiological factors and their translational impact on clinical detection, diagnosis, and treatment of PAD. Through our critique, we reveal some of the advantages and shortcomings of imaging modalities, explore the translational potential of non-invasive imaging in small animals to humans, and summarize future research directions in non-invasive imaging diagnostics and therapy. We will explore the uses of ultrasound, near infrared imaging (NIR), particle image velocimetry, radionuclide imaging, magnetic resonance imaging, computed tomography, optical imaging, positron emission tomography, and photoacoustic tomography in small animals. The following sections are organized by PAD imaging in the: (1) aorta; (2) carotid and cerebrovascular system; and (3) lower limb peripheral vasculature. For ease of reference, Table 1 lists the acronyms used in this review.

**Table 1:** Acronym Definitions.

Acronym	Definition	Acronym	Definition
^18^F-FDG	^18^F-Fluorodeoxyglucose	MCA	Middle Cerebral Artery
AAA	Abdominal Aortic Aneurysm	MMP	Matrix Metalloproteinase
ADC	Apparent Diffusion Coefficient	MRA	Magnetic Resonance Angiography
Alk1	Activin receptor-like kinase 1	MRI	Magnetic Resonance Imaging
AngII	Angiotensin II	MSC	Mesenchymal Stem Cell
apoE^−/−^	Apolipoprotein E-deficient	NIR	Near Infrared
AVM	Arteriovenous Malformation	NIRF	Near Infrared Fluorescence
B-mode	Brightness Mode	NO	Nitric Oxide
BM-MNC	Bone-Marrow-derived Mononuclear Cell	PAD	Peripheral Artery Disease
BOLD	Blood-Oxygen-Level Dependent	PET	Positron Emission Tomography
CAD	Coronary Artery Disease	PW Doppler	Pulsed Wave Doppler
CEUS	Contrast-enhanced Ultrasound	PWI	Perfusion Weighted Imaging
CLI	Critical Limb Ischemia	rmVEGF	Recombinant murine Vascular Endothelial Growth Factor
CT	Computed Tomography	rt-PA	Recombinant Tissue Plasminogen Activator
CTA	Computed Tomography Angiography	SAH	Subarachnoid Hemorrhage
DE	Delayed-enhancement	smLRP1^−/−^	Smooth Muscle Specific Low-density Lipoprotein Receptor-related Protein 1-deficient
ECG	Electrocardiogram	SPECT	Single Photon Emission Computed Tomography
eNOS	Endothelial Nitric Oxide Synthase	TAA	Thoracic Aortic Aneurysm
HHT	Hereditary Hemorrhagic Telangiectasia	TOF-MRA	Time-of-flight Magnetic Resonance Angiography
LDLR^−/−^	Low-density Lipoprotein Receptor-deficient	US	Ultrasound
LDPI	Laser Doppler Perfusion Imaging	VCAM-1	Vascular Cell Adhesion Molecule 1
M-mode	Motion Mode	VEGF	Vascular Endothelial Growth Factor

### 1.1. Ethics and Regulations of Animal Models

While animal models are important tools in investigating disease diagnosis, development, and potential treatments, their use comes with great responsibility. There are guidelines that are observed to ensure humane treatment of animals, including those outlined by American Association for Laboratory Animal Science (AALAS), National Center for Replacement, Refinement, and Reduction of Animals in Research (NC3Rs), and individual Institutional Animal Care and Use Committees (IACUC), focusing on improving animal care and minimizing suffering [3]. Additionally, the Association for Assessment and Accreditation of Laboratory Animal Care (AAALAC) provides voluntary institutional accreditation and assessment programs to demonstrate fulfillment of minimum standards required by law. AAALAC accreditation involves a site visit investigating the institution’s animal care and use program including management and oversight, veterinary care, and facilities. IACUC oversight, while varying slightly between institutions, involves the approval of animal experimental procedures and verification of personnel animal training. Refinement efforts help ensure that animals are provided good living conditions, pathogen-free environments, ample food and water, and are properly euthanized. If possible, rodents should also be provided with environmental enrichment, such as toys, social housing, nesting material, and other measures to improve quality of life.

Computer simulations, mathematical models, or *in vitro* models encourage methods that help minimize animal usage, following replacement and reduction efforts. In some cases, computer simulations of pathophysiological disease processes may be adequate and could be used to replace *in vivo* studies [4,5]. These simulations may become more appropriate as modeling becomes more advanced and accurate. Additionally, by combining animal models and *in vivo* imaging, we can effectively decrease the number of animals needed for experimental study. In this review, we briefly discuss the emerging use of computer simulations in conjunction with *in vivo* imaging of small animal PAD models.

### 1.2. Pathophysiology of Atherosclerosis

**Figure 2:**
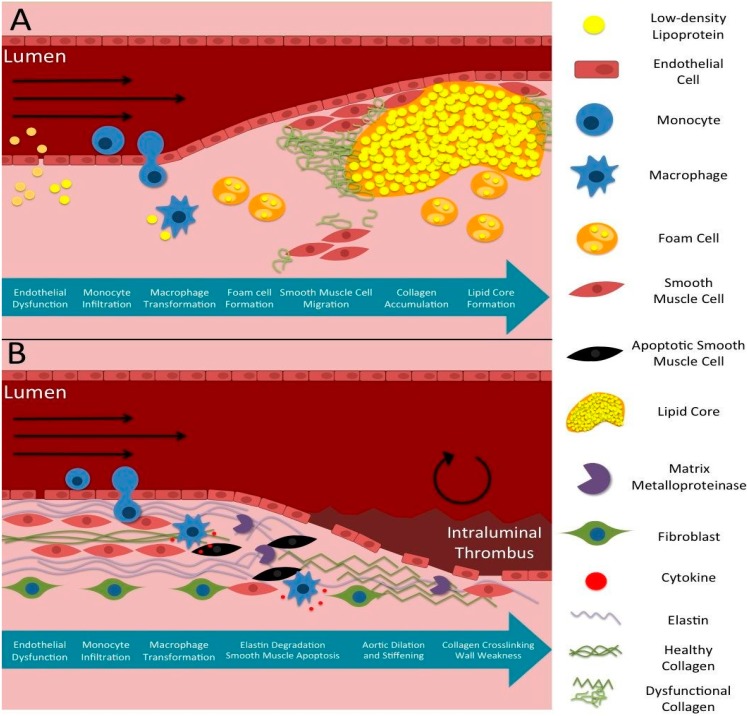
Typical Disease Progression of Atherosclerotic and Aneurysmal Disease. (**A**) Chronological atherosclerotic plaque formation starting at endothelial dysfunction and ending in lipid core formation and turbulent, reduced blood flow; (**B**) Chronological aneurysm formation highlighting similar early steps, but resulting in extracellular matrix degradation, vessel expansion, and turbulent flow.

Atherosclerosis is a localized arterial disease caused by plaque buildup in the intimal layer of arteries, often developing in the aorta, carotid, coronary, and peripheral arteries (Figure 2A; [6]). In the early stages of plaque development, monocytes migrate into the subendothelial space, transform into macrophages, and begin to take up lipids. As a result, macrophages transform into foam cells and accumulate to form arterial plaque [7,8]. Smooth muscle cells migrate from the medial layer to the intima and produce collagen that further increases plaque progression and fibrous cap formation. Additionally, low wall shear stress and flow velocity, as well as dysfunctional flow profiles at certain parts of vessels, such as bifurcations, have been shown to promote atherosclerosis progression [9,10]. Plaques can also be categorized as stable or vulnerable depending on their structural characteristics. Stable plaques are less likely to rupture because their calcification contributes more to outward vessel remodeling [6]. Vulnerable plaques, characterized by their thin fibrous cap over a large lipid and macrophage core [8], are mostly rupture-prone and thus are frequently a cause of thrombotic or embolic complications such as ischemic stroke and myocardial infarction [7,8]. In such plaques, matrix metalloproteinases (MMPs) further degrade the endothelium and cause smooth muscle cell death [7,8], while macrophages within the fibrous cap secrete proteases that promote collagen and peptide degradation; all these factors lead to an increased likelihood of rupture [8]. Furthermore, hemodynamic forces have also been shown to contribute to plaque destabilization [9]. Overall, atherosclerosis is a multifactorial disease that progresses by both hemodynamic forces and pro-atherosclerotic factors.

Atherosclerosis develops systemically with varying symptoms specific to the location of plaque development and has unique risk factors such as diabetes mellitus, dyslipoproteinemia, and familial hypercholesterolemia [11]. Atherosclerosis in coronary arteries is a major cause of myocardial ischemia; similar progression in the extracoronary circulation also has serious implications, such as ischemic strokes caused by emboli from ruptured carotid plaques [11]. Although manifestations of coronary artery disease (CAD) and PAD both stem from systemic atherosclerosis, the etiologies of these diseases are multifactorial. Hence, despite documentation that patients with PAD also have coronary atherosclerosis [12,13,14], it is also not uncommon for CAD to occur without any concomitant PAD or vice versa [15]. So far, the mechanisms by which the progression of PAD and coronary atherosclerosis differ remain to be elucidated. Some studies, however, have put forth that differences in progression could be attributed to initiating factors for atherosclerosis [16], genetic variations [17], demographic, smoking status, inflammatory state, and serum lipoprotein levels [18].

Non-invasive imaging techniques have been utilized to help investigate the differences in CAD and PAD progression [19]. For example, by characterizing the progression of coronary atherosclerosis with intravascular ultrasound in patients with lower limb athero-thrombosis, researchers observed more extensive and calcified coronary atherosclerosis and impaired arterial remodeling. Such results indicate that coronary atherosclerosis among patients with lower limb athero-thrombosis could be a result of constrictive remodeling of the coronary arteries [20]. Nonetheless, more studies are needed to explore the specific differences between coronary and extracoronary atherosclerosis progression.

Atherosclerosis-induced lower limb ischemia, which we will refer to as lower limb PAD, oftentimes leads to poor blood flow to the lower limbs due to arterial stenosis or thrombosis. As a consequence, lower limb PAD commonly manifests as intermittent claudication and critical limb ischemia in later stages of disease progression [21]. Data from the Framingham cohort showed that 20% of symptomatic patients with lower limb PAD also had diabetes mellitus [22]. However, the risk of developing lower limb PAD also increases with obesity, previous history of other atherosclerotic disease [23,24], high triglycerides, low high-density lipoprotein, and aging [25].

### 1.3. Pathophysiology of Aneurysms

Aneurysms are arterial dilations of at least 1.5 times the size of the healthy vessel diameter, often exacerbated by high blood pressure expanding weakened arterial walls (Figure 2B; [26]). They also involve vessel stiffening, proteolytic degeneration in vessel walls, inflammation, and can have unique characteristics and complications corresponding to their location in the aorta or cerebral vasculature. Thoracic aortic aneurysms (TAAs) are typically dissecting aneurysms in the aortic arch, while abdominal aortic aneurysms (AAAs) are usually fusiform aneurysms forming below the kidneys. Cerebral aneurysms arise most commonly near bifurcations in the cerebral vasculature, with most occurring at the anterior (30%) and posterior (25%) communicating arteries [27]. They may sometimes develop from cerebral arteriovenous malformations (AVMs), abnormal connections between cerebral arteries and veins, but the relationship is not well defined [28]. Cerebral AVMs create a low-pressure shunt, diverting blood flow from the arterial side to the venous side, which can cause complications from recurrent bleeding, vascular rupture [29], or reduced cognitive function due to ischemia [30,31]. Both aneurysms and AVMs can grow and eventually lead to vessel rupture and internal bleeding, causing systemic hypotension in aortic aneurysm rupture or hemorrhagic stroke in cerebral aneurysm or AVM rupture.

Atherosclerosis can sometimes contribute to the pathophysiology of hemorrhagic cerebrovascular aneurysms because it initiates endothelial dysfunction, catalyzing a signaling cascade that weakens the vessels and causes aneurysm formation [32,33]. Other factors including polycystic kidney disease, α1-antitrypsin deficiency, increased levels of plasma elastase, and altered MMP activity, can also affect the mechanical integrity of vessel walls [32,33]. Elastase and MMP normally dictate tissue repair, vascular remodeling, and the maintenance of the extracellular matrix [32,33]; however, inappropriately elevated serum levels of elastase and MMP compromise the native composition of the arteries, locally weakening the vessel wall and subsequently resulting in an increase in the vessel’s aspect ratio [32,33].

### 1.4. The Clinical Need for Early Diagnosis of PAD

A significant proportion of the United States population suffers from fatal complications due to atherosclerosis, aneurysms, and cerebral AVMs. For example, stroke accounts for nearly 25% of total deaths [6], with 795,000 new cases annually, 185,000 of which are recurring [11]. Lower limb PAD caused by athero-thrombosis is the third leading cause of atherosclerosis morbidity after stroke and coronary heart disease [34]. Due to the fact that the majority of lower limb PAD patients are asymptomatic, the overall prevalence might be underestimated [1]. In the United States, approximately 1%–5% of the adult population have cerebral aneurysms [27] that can lead to subarachnoid hemorrhage (SAH) accounting for approximately 1% to 7% percent of strokes annually [35,36]. In the United States, 9000 cases of TAA were reported in 2007 [37], and the latest estimates suggest that 300 million people globally have AAAs with increasing prevalence in the developing world [26]. AVM occurs in approximately 18 out of every 100,000 Americans and accounts for one-third of intracerebral bleeding in young adults 20 to 40 years old [38]. Like other PAD, AVMs often do not present clinical symptoms. For example, even at the time of detection, at least 15% of people affected by AVMs are asymptomatic [38]. Because of the complexity of the disease, the multitude of factors that contribute to a normally asymptomatic disease, and the discrepancies of current imaging methods, there is an unmet clinical need for accurate imaging modalities with early disease detection abilities.

Current projections estimate that many will continue to suffer from PAD complications in the future. It has been suggested that in the United States, 795,000 new cases of stroke will occur every year, and by 2050, stroke cases will more than double, particularly among those ages 75 and older and in minority populations [39]. There is also data suggesting that 45 of the 202 million patients with lower limb PAD will die from heart or cerebrovascular disease within a 10-year period [34]. Delayed diagnosis and inaccurate evaluation of PAD disease progression may lead to poor prognostic outcomes. For example, SAH leaves the majority of patients with neurological or cognitive impairment [35,36], and AAA rupture leads to sudden death. Taken together, these statistics highlight the prevalence of PAD and suggest that early detection and evaluation of disease progression is paramount in combating PAD morbidity and mortality.

### 1.5. Non-Invasive Imaging Strategies in Small Animals

Non-invasive and dynamic small animal imaging techniques promote the translational potential of novel technology currently applied in bench research. Importantly, longitudinal studies are reducing the need to sacrifice a large number of animals [40]. Some common imaging modalities are described below. Table 2 compares advantages/disadvantages of different imaging modalities with respect to small animal PAD models.

**Table 2:** Non-invasive Imaging in Small Animal PAD Models.

Imaging Modality	Advantages	Disadvantages	Cardiovascular Imaging Contrast Agents
Ultrasound	Fast acquisition time	Acoustic artifacts Often difficult to interpret	Microbubbles
	Portable		
	High spatiotemporal resolution		
	No harmful radiation		
Magnetic Resonance Imaging	Non-ionizing	Lower temporal resolution compared to ultrasound Difficult to time bolus injections of contrast agents High cost	Gadolinium-based contrast agents Iron oxide and other paramagnetic particles
	Superior tissue differentiation		
	Provides anatomical, functional, and molecular information		
	Whole body imaging capability		
Positron Emission Tomography	Provides quantitative pharmacokinetic information on radiotracer distribution throughout the body	Requires radioactive contrast agents with short half-lives	^11^C, ^18^F, ^13^N, ^15^O, ^82^Rb
		Limited spatial resolution (1–2 mm)	
Single-Photon Emission Computed Tomography	Provides molecular and functional parameters	Dependent on the pharmacodynamics and kinetics of the tracer	^99m^Tc, ^111^In chelates
	No depth limitation		
Computed Tomography	Fast acquisition time	Ionizing radiation Lacks soft tissue differentiation May require contrast agent	Iodine Barium
	High spatiotemporal resolution		
	Provides information about the spatial geometry, luminal patency, and vascular networks		
Diffuse Optical Imaging	High sensitivity	Limited depth of penetration (1–2 mm) Susceptibility to photobleaching	Fluorophores Luciferin/luciferase
	No ionizing radiation		
	Availability		

#### 1.5.1. Magnetic Resonance Imaging

Magnetic Resonance Imaging (MRI) is a non-ionizing diagnostic imaging modality that uses strong magnetic fields and radiofrequency pulses to reconstruct high-resolution images of anatomical structures. The majority of MRI techniques quantify the relaxation rates of protons excited by changes in magnetic pulses. There are also methods that allow users to obtain hemodynamic and structural information from an animal. Contrast-enhanced MRI typically requires T1 and T2 contrast agents [41], creating alterations in the relaxation properties of blood [42,43,44], atherosclerotic plaque [45], and aneurysms [46,47]. Magnetic Resonance angiography (MRA) can be used to visualize vascular structure in small animals [48,49]; however, the fast heart rate and small blood volume of rodents often make it difficult to time bolus injections of gadolinium-DTPA MRI contrast agents [42]. Non-contrast MRI is also used to characterize hemodynamics of the vasculature. Examples include: (1) time-of-flight MRA (TOF-MRA) to highlight blood movement relative to static tissue [50]; (2) phase-contrast MRA to measure temporally and spatially resolved blood flow velocity [51,52]; and (3) arterial spin-labeling where flow is excited proximal to the region of interest [53]. Although typically performed with magnets at lower field strengths, clinical application of TOF-MRA techniques is useful for monitoring and pre-surgical imaging [54]. Furthermore, blood-oxygen-level dependent contrast (BOLD) imaging is a powerful tool for investigating changes in oxygenation levels [55,56]. All of these techniques highlight the utility of MRI as a non-ionizing imaging modality capable of investigating multiple aspects of small animal vasculature.

#### 1.5.2. Computed Tomography

Computed Tomography (CT) is a diagnostic anatomical imaging modality that reveals internal structures through the detection of X-rays transmitted through the body, given that X-ray absorption varies based on tissue densities. X-ray projections at different angles and multiple slices are acquired and reconstructed to create a 3D image. While CT has high spatial and temporal resolution, it lacks the superior soft tissue differentiation achieved by other methods such as MRI [57,58]. The benefit of using contrast-enhanced CT angiograms (CTA) is that it can provide information about the spatial geometry, luminal patency, and connections within vascular networks. Researchers are applying micro-CT angiography [59,60,61,62] for visualization of vessel anatomy and gross morphology, commonly with a vascular contrast agent [63,64].

#### 1.5.3. Ultrasound

Ultrasound (US) is a common anatomical imaging modality using acoustic waves emitted from a transducer to produce images of internal structures based on acoustic reflections. US can also be used to measure biomechanical parameters such as blood velocity using Doppler imaging and vessel wall motion using motion mode (M-mode) imaging. The major advantages of US are the lack of ionizing radiation, fast imaging speed, and portability without the need for special shielding, as is required with MRI. High frequency systems capable of anatomical or brightness mode (B-mode) imaging and Doppler US offer high temporal and spatial resolution and have been developed specifically for use in small animal research. Additionally, contrast-enhanced ultrasound (CEUS), which uses microbubbles as echogenic contrast agents, allows for microvasculature imaging [65] and molecular targeting of biomarkers [66].

#### 1.5.4. Optical Imaging

Laser Doppler perfusion imaging (LDPI) non-invasively detects blood perfusion in the microvasculature. As the name suggests, LDPI is an application of Doppler shift where moving particles, such as red blood cells, produce a change in laser frequency. This shift can be detected and analyzed to produce a color map of blood flow. LDPI is one of the most widely used imaging modalities in lower limb PAD research; however, LDPI has low intrinsic depth of penetration and therefore estimates of skeletal muscle perfusion or other deeper tissue imaging are limited [67]. Hyperspectral imaging is another type of optical imaging that has recently been applied to quantify oxygen saturation levels in small vessels close to the skin [68]. By this method, one acquires a series of images across a narrow spectral band, ranging from 500 to 660 nm, to measure oxygen saturation levels [68,69]. As oxyhemoglobin and deoxyhemoglobin have unique absorbance and reflectance properties, hyperspectral imaging can detect local spectral properties and quantify oxygen concentration levels in the bloodstream [69]. Near-infrared fluorescence (NIRF) imaging is another type of optical imaging that uses injectable fluorescent contrast agents, excited by NIR light in the 700–900 nm range, for targeted detection of molecular markers while minimizing the background tissue autofluorescence. Although it is limited in depth penetration, it is advantageous because NIR wavelengths have low tissue absorption, and the emitted light has high molecular sensitivity [70].

#### 1.5.5. Positron Emission Tomography and Single Photon Emission Computed Tomography

Positron Emission Tomography (PET) and Single Photon Emission Computed Tomography (SPECT) are molecular and functional imaging modalities that utilize radioactive tracers to evaluate healthy and diseased physiology in the body. The tracers, which may be swallowed or injected, target cell receptors throughout the body or accumulate in regions with high metabolic activity. Through detection of the emitted gamma rays, the kinetics and distribution of the tracer can be determined and used to reconstruct 3D images. PET and SPECT have advantages in being highly quantitative and targeted techniques; however, their utility is dependent on the pharmacodynamics and kinetics of the tracer. In preclinical research, micro-PET and SPECT have been developed, some in combination with CT, but are limited by spatial resolution and motion [71].

### 1.6. Advances in PAD Imaging

Recent research is benefiting from advances in existing and emerging small animal imaging techniques in multiple ways. Since the morphology and heterogeneity of atherosclerotic lesions and aneurysms are key to understanding these conditions, imaging techniques capable of characterizing evolving vascular structure and composition, including changes in elastin, collagen, fibrin, and lipid content, have become a recent focus [72,73,74]. Also, hemodynamic changes in diseased vessels contribute to PAD pathogenesis [75,76,77], and several advanced biomechanical metrics may be more sensitive than current diagnostic techniques [78]. Finally, molecular imaging aimed at quantifying biomarkers targeted to specific cell types or pathways are helping to determine the role metabolism, inflammation, and growth factors play in PAD progression [79,80,81]. All of these advancements in imaging technology are aimed at providing researchers with the ability to serially track PAD as it develops. The sections below highlight three emerging areas that are being used in PAD research.

## 2. Aortic Disease

### 2.1. Small Animal Models of Aortic Disease

Preclinical aortic aneurysm [82,83] and atherosclerosis [84] research rely on small animal models to investigate disease progression. The vast majority of models fall into two primary categories: chemically-induced and genetically-induced. This section describes several of the most common aortic disease models that include atherosclerosis and either TAAs or AAAs.

Two primary chemically-induced AAA models are the calcium chloride (CaCl_2_) [85] and the porcine pancreatic elastase [86] models. The CaCl_2_ AAA model requires the application of CaCl_2_ to the periadventitial surface to induce inflammatory cell infiltration in the infrarenal aorta [85]. This model causes disruption to the targeted elastin network with calcium precipitates, activating the inflammatory response [85]. It also models aortic calcification and vascular smooth muscle cell apoptosis, but does not bring about thrombus or aortic rupture as seen in human AAA progression [87]. The elastase model, on the other hand, induces an initial mechanical expansion due to pressurized, localized intraluminal perfusion of elastase solution [86,88]. Subsequently during progression, elastin fibers degrade and MMP activity in the vessel wall increases. This model is clinically significant as the elastase solution induces inflammatory cell recruitment that can mimic the degeneration of infrarenal aortic elastin fibers [89]. One common variation of this model is to use periadventitial elastase application [90] similar to the CaCl_2_ model.

Genetically engineered small animal models are also used to study the development of aortic disease, often in apolipoprotein E-deficient (apoE^−/−^) [91,92] or low density lipoprotein receptor-deficient (LDLR^−/−^) [93] hyperlipidemic mice. ApoE^−/−^ mice develop atherosclerosis spontaneously as they age, and plaque development is enhanced and accelerated by a high fat diet [94,95]. The apoE^−/−^ model is often combined with continuous infusion of angiotensin II (AngII) that increases systemic blood pressure and leads to dissecting suprarenal AAAs [93,96]. The primary features of this model include inflammatory cell recruitment, transmural hematomas, and atherosclerotic plaque build-up [97]. Although an inflammatory component is also evident in both the CaCl_2_ and elastase models, coexisting atherosclerosis, suprarenal dissection, and potential for aortic rupture are exclusive features of this model. Recently, AngII infusion has also been shown to exacerbate superior mesenteric arterial and ascending aortic dilation in smooth muscle cell specific low-density lipoprotein receptor-related protein 1 deficient (smLRP1^−/−^) mice [98]. The AngII smLRP1^−/−^ model exhibits minimal macrophage accumulation, but significantly increased elastin degradation [98]. Other uses of engineered genetic manipulations include the fibulin-4 mouse model for TAAs [99]. Mice with reduced expression of fibulin-4 show a mild phenotype involving occasional small aneurysm formation, which more closely mimic sporadic and difficult to detect human TAAs [99].

These aneurysm models do not perfectly replicate human TAAs or AAAs. However, experimental *in vivo* imaging studies may help to better elucidate disease mechanisms, understand pathogenesis, and evaluate potential therapeutics using a combination of models [100]. More specifically, any apparent association between aortic aneurysms and atherosclerosis [101] could be better studied with more advanced non-invasive structural, compositional, and molecular imaging techniques.

### 2.2. Anatomic and Biomechanical Measurements

In clinical practice, maximum aortic diameter is used to determine the size of AAAs (typically defined as 3.0 cm or greater [102]) and is often first detected by abdominal palpation, US, or CT. Monitoring changes in aortic diameter using CT [103,104] or US [105] sometimes lacks sensitivity when vessel expansion is subtle or the aneurysm has a complex geometry [106]. For this reason, researchers are interested in exploring the utility of more advanced imaging-derived measurements of AAAs. Small animal disease models have become an important part of this image development process.

**Figure 3:**
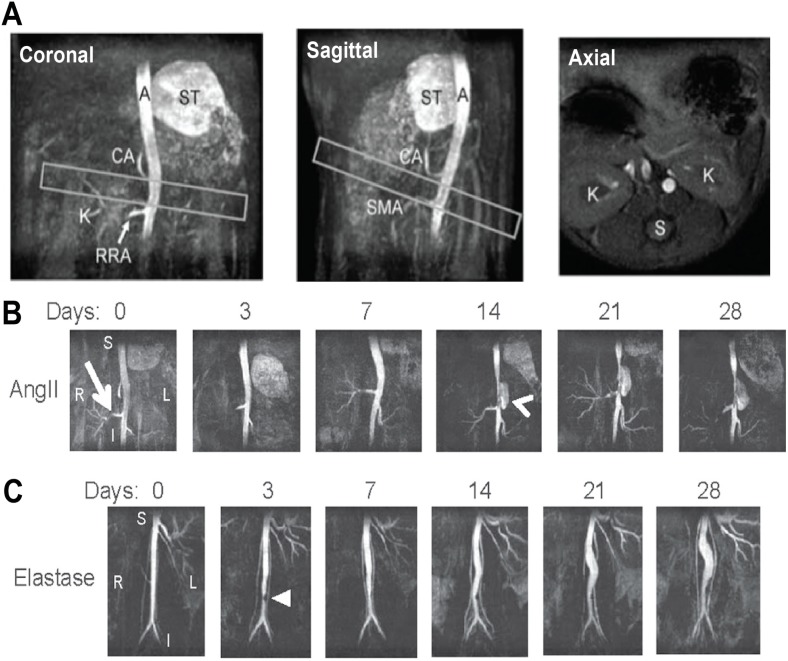
Time-of-flight Magnetic Resonance Angiography (TOF-MRA) of Abdominal Mouse Aorta with and without Aneurysm. (**A**) TOF-MRA images of a healthy mouse aorta are shown as maximum intensity projections. Leftward position and antero-posterior curvature above the kidneys can be seen (boxes). The aorta (A), celiac artery (CA), kidney vasculature (K), right renal artery (RRA), spine (S), stomach (ST), and superior mesenteric artery (SMA) are labeled for landmark identification; (**B**,**C**) Coronal TOF-MRA maximum intensity projections show lumen expansion in (**B**) AngII and (**C**) elastase AAAs over 28 days. The AngII aneurysm appears suddenly (arrowhead) above the right renal artery (arrow) and expands leftward. The elastase aneurysm expands slowly, and a small region of signal hypointensity is observed due to a suture in the vessel at day 3 (triangle). Subfigure A is adapted with permission from *Goergen et al.* [108]. Subfigures **B** and **C** are adapted with permission from *Goergen et al.* [50].

*Turner* *et al.* [107] first demonstrated the sensitivity of small animal MRI to track AAA progression non-invasively and to monitor treatment effects. Using the AngII apoE^−/−^ mouse model, *Goergen* *et al.* [50] found a strong correlation between aortic expansion and increased vessel motion and aortic curvature. This is in agreement with their previous findings [108] showing that vessel motion is leftward in the suprarenal aorta where there is large aortic curvature (Figure 3A). Using TOF-MRA, they observed that volumetric expansion increased as cyclic strain decreased in both AngII apoE^−/−^ and elastase AAA mice (Figure 3B) [50]. Overall, these results reinforce the significance of aortic geometry and motion on the progression of experimental AAAs. Combined with other studies linking hemodynamics to AngII aneurysm formation [109], such findings highlight the importance of biomechanics on both aneurysm initiation and progression.

**Figure 4:**
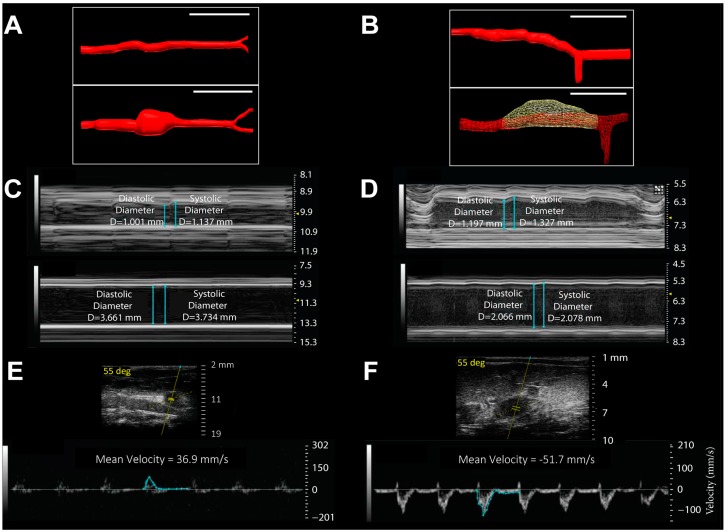
Rodent Models of Abdominal Aortic Aneurysms Exhibit Significant Changes in Volume, Circumferential Cyclic Strain, and Blood Flow Velocity. (**A**,**B**) Aortic volumes increase over 28 days in (**A**) elastase rats and (**B**) AngII apoE^−/−^ mice. 3D B-mode renderings are shown from before (**top**) and after (**bottom**) AAA formation. Scales: (**A**) 10 mm and (**B**) 5 mm; (**C**,**D**) Circumferential cyclic strain decreases over 28 days in (**C**) elastase rats and (**D**) AngII apoE^−/−^ mice. M-mode tracings are shown from before (**top**) and after (**bottom**) AAA formation. Scale: depth in mm; (**E**,**F**) Mean aortic blood flow velocity decreases after 28 days. Representative velocity waveforms on long-axis PW Doppler at the site of AAAs in an (**E**) elastase rat and (**F**) AngII apoE^−/−^ mouse. Baseline blood velocity values are 342.1 and 461.2 mm/s, respectively. Adapted with permission from *Phillips et al.* [112].

Researchers are also using high frequency US to measure aortic expansion and hemodynamic changes in experimental AAAs. Using similar M-mode US aortic imaging [50,110], *Favreau et al.* [111] and *Phillips et al.* [112] found that AAAs displayed large reductions in cyclic strain (Figure 4B). *Phillips et al.* [112] further demonstrated the ability to track and quantify volumetric expansion and blood flow velocity using 3D B-mode and Pulsed Wave (PW) Doppler US, respectively, in AngII apoE^−/−^ and elastase AAAs (Figure 4A,C). *Luo et al.* [113] have also observed that the interaction of wall motion in AAAs with blood flow is irregular, likely due to regional stiffening and vessel wall inhomogeneities. This dual approach utilizing information of blood velocity and vessel wall velocity may provide important parameters for future biomechanical modeling. With continuing research on biomechanical changes in AAAs, aortic strain and flow measurements using US may also provide useful metrics for predicting AAA growth in humans [114,115].

Recent work has also begun using computational flow dynamics to model aortic disease in mice [64,116,117]. Vortex shedding is the periodic detachment of vortical flow structures when the flow undergoes a sudden expansion, a phenomenon that occurs when blood flows through an aneurysm. Small animal US with high temporal resolution ( Supplementary Video 1) and with electrocardiogram (ECG)-gating (Supplementary Video 2) can provide much higher frame rates sufficient for visualizing complex flow patterns and vessel motion. Piomelli and colleagues observed that ECG-gated B-mode and Power Doppler US reveal high levels of vortex shedding and fluctuating wall shear stress in the AngII apoE^−/−^ model and related these findings to suprarenal dissection in the model [118,119]. This work shows that the vortex shedding occurs at the same location in both mice and humans, making it susceptible to rupture, but the key difference is that it occurs during systolic acceleration in mice and during diastolic deceleration in humans. These novel metrics represent an important frontier for studying aortic disease pathogenesis.

### 2.3. Novel Molecular Imaging of Aortic Disease

The application of molecular imaging to aortic disease detection and treatment monitoring is a relatively new area in this field. With a growing range of potential biomarkers for AAAs and atherosclerosis, molecular targets would be useful in clinical imaging. To date, clinicians have been primarily interested in the ability of ^18^F-fluorodeoxyglucose (^18^F-FDG), the most commonly used metabolic radiotracer, to measure aortic inflammation in AAAs [120,121,122] and atherosclerosis [123]. Some clinical studies, however, have challenged the notion that ^18^F-FDG uptake in AAAs is sufficient for determining disease progression [124,125].

In small animal models of AAAs, several different molecular imaging targets are being investigated. *English* *et al.* [126] recently found that ^18^F-FDG could predict aortic rupture in elastase rats infused with β-aminopropionitrile, a rupture-inducing agent. *Klink et al.* [46] have suggested that collagen-targeted nanoparticles may discriminate non-expanding and rupture-prone AAAs using molecular MR with a rupture model involving infusion of AngII and anti-transforming growth factor-β in wild type mice [127]. Using elastase-induced murine AAAs with intraluminal thrombus formation, *Sarda-Mantel et al.* [128] found a correlation in the uptake of an apoptosis-targeted radiotracer in these areas with thrombi in harvested *ex vivo* human AAAs. Using either radiolabeled peptides or nanoparticle platforms, other groups have targeted and measured tracer uptake in macrophages or vascular smooth muscle cells in the AAA wall of AngII apoE^−/−^ mice, highlighting the ability to measure AAA inflammation [129,130] and neovascularization [129]. Furthermore, Botnar and colleagues have used an elastin-targeted MR agent to localize and track vessel wall elastin degradation and repair in AngII apoE^−/−^ mice before and after aortic expansion [47].

Few non-invasive imaging studies have been published to date that use the CaCl_2_ or fibulin-4 model. To further quantify the inflammatory processes that drive AAA development, *Sheth et al.* [131] used *in vivo* optical molecular imaging to measure MMP activity in the walls of CaCl_2_-induced AAAs. With these activatable optical probes, they demonstrated a linear relationship between proteolytic activity and aneurysmal growth [131]. The ability to predict the rate of aneurysm growth could help guide medical and surgical management of AAAs. *Kaijzel et al.* [99] demonstrated that NIRF imaging of MMP in mice underexpressing fibulin-4 reveals the site of TAA formation in the aortic arch and subsequent macrophage accumulation. The ability to predict the exact site of TAAs could be important for future preclinical *in vivo* imaging studies requiring early stage disease diagnosis.

Atherosclerosis models, on the other hand, have been more extensively investigated with molecular imaging. Characterization of inflammatory progression in apoE^−/−^ mice [132] and aortic remodeling in Watanabe rabbits, a model of heritable hypercholesterolemia [133], has been possible using ultrasmall superparamagnetic iron oxide particles and gadolinium-based agents, respectively. Several groups have also developed novel targeted nanoformulations [134], such as phospholipid-containing liposomes, for tracking macrophages in atheromas with the Watanabe rabbit model [135,136]. Targeting hypoxic factors may be another possible means for localizing aortic plaques containing macrophages and neovessels [137].

**Figure 5:**
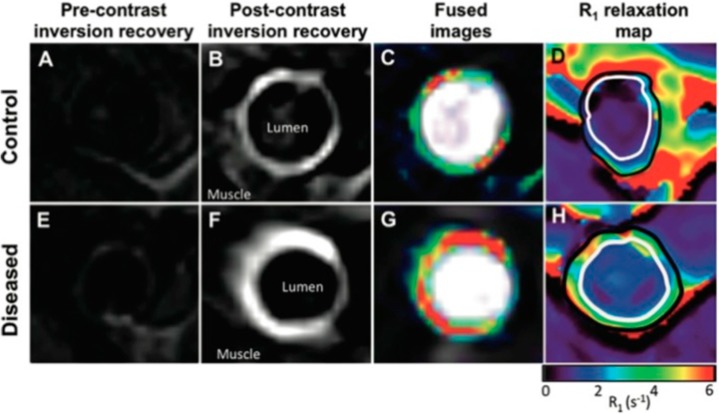
MRI of Aortic Cross-sections Show Higher Uptake of Elastin-Specific Contrast Agent and R1 Relaxation Values in Atherosclerotic Compared with Control Aortas. (**A**,**E**) Phase-contrast MRA pre-injection and (**B**,**F**) delayed-enhancement (**D**,**E**) MRI post-injection of elastin-specific contrast agent; (**C**,**G**) DE MRI fused with phase-contrast angiograms; (**D**,**H**) Corresponding R1 relaxation maps post-injection of elastin-specific contrast agent showing less relaxation in the atherosclerotic aorta. Reproduced with permission from *Phinikaridou et al.* [139].

Other ongoing research efforts seek to sensitively detect vulnerable plaques in hyperlipidemic models. The use of a radiolabeled MMP-inhibitor [138] and an elastin-targeted MR agent (Figure 5, [139]) are two notable examples. In the latter example, *Phinikaridou et al.* [139] observed weak MR signal enhancement in aortas pre-contrast using phase-contrast MRA (Figure 5A,E). However, there was good correlation between elastin area with signal area on delay-enhanced MR images in control (Figure 5B,C) and atherosclerotic (Figure 5F,G) rabbit aortas after injection of the elastin-targeted agent. These areas of signal enhancement included expected elastin accumulation in the neointima of atherosclerotic aortas. Moreover, their post-contrast MR data provided quantitative relaxation maps (Figure 5D,H) showing a significantly decreased relaxation rate of the atherosclerotic aortic walls [139].

Taken together these findings strengthen the evidence that molecular-level compositional methods can be used to identify areas of localized inflammatory cell or macromolecule accumulation in diseased vasculature. Translational potential for diagnostic agents however will depend on composition, size, and targeting ability of the contrast agent. For PAD with aortic disease patients, two of the most useful applications of these methods are in disease staging and risk assessment for plaque rupture or aortic vessel rupture. Furthermore, novel therapeutic strategies, such as microRNA delivery [82,140], could be non-invasively monitored using small animal imaging and accelerate the translation of these therapies.

## 3. Carotid and Cerebrovascular Disease

### 3.1. Technologies for Carotid and Cerebrovascular Imaging

CT and MRI are widely used in the clinic to detect cerebrovascular disease, including cerebral aneurysms, ischemic strokes, and hemorrhagic strokes. They can indirectly detect the consequences of vascular disease as white matter lesions [141] and directly detect vascular abnormalities using structural imaging methods such as CT or MR angiography. However, these methods tend to have low specificity for pathophysiological diagnosis, providing an incomplete understanding of disease progression. The understanding of cerebrovascular pathology could be improved with small animal non-invasive imaging focused on identifying potentially new therapies or technologies that can guide treatment decisions in the clinic. Furthermore, small animal models are well suited for studying cerebrovascular diseases because of anatomic similarities in the cerebral vasculature of rodents compared to humans [61]. In the following section, we will discuss recent advancements in our understanding of pathophysiology of carotid atherosclerosis, aneurysms, and AVMs using non-invasive imaging and address their translational potential.

### 3.2. Carotid Atherosclerosis and Cerebral Ischemia

#### 3.2.1. Small Animal Models of Carotid Stenosis and Cerebral Ischemia

Several different small animal models of cerebral ischemia and carotid stenosis have been developed to study stroke. These include models that create cerebral thrombus by laser-induced clotting [142], ferric acid [143], or electricity [144] applied to the carotid artery in rodents. These models require a surgical procedure that damages the artery to initiate thrombi, which adds confounding factors. A recent improvement developed by *Yeh et al.* [145] involved the implantation of a light emitting diode next to the carotid artery followed by an injection of photosensitive dye that can be hardened at a controlled rate. Carotid emboli, in contrast, have traditionally been created by inserting a length of suture down the carotid artery to block flow in the middle cerebral artery [146,147]. In an effort to match the natural development of carotid stenosis leading to an embolism, *Schunke et al.* [148] pioneered a technique that used targeted collagen injections into the carotid arteries of rats, producing a gradual luminal narrowing that slowly disrupted arterial flow and eventually produced emboli. Other models have focused on dispersed particle deposition with traceable microspheres to cause blockages downstream that mimic embolic particle dispersions [149]. Novel rodent models for carotid atherosclerosis have also been developed to mimic progressive arterial stenosis. One of these stenosis models utilizes a hydrogel cuff that closes at a specified rate around the carotid artery [150], closely mimicking advanced atherosclerosis. Likewise a suture tie technique developed by *Tao et al.* [151] uses a simple surgical method, demonstrating a chemical free, adaptable mechanical stenosis model. All of these small animal models mimic important aspects of carotid atherosclerotic disease, thus allowing detailed investigation of disease progression.

#### 3.2.2. Structural and Hemodynamic Imaging of Carotid Stenosis and Ischemic Stroke

**Figure 6:**
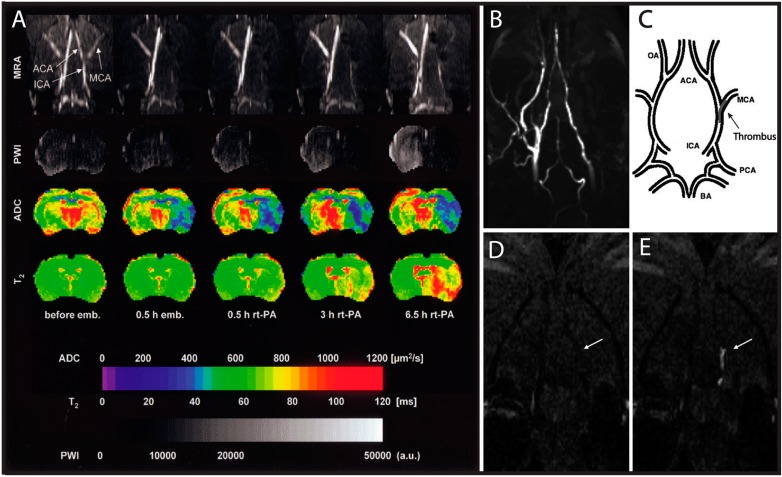
MRI of Thromboembolic Stroke in a Rat Model. (**A**) Multiparametric MRI of rat brain before and after intracarotid clot embolism treated at 1 h with intra-arterial infusion of recombinant tissue plasminogen activator (rt-PA). MRA of horizontal maximum-intensity projection maps focusing on the Circle of Willis; PWI, perfusion-weighted imaging; ADC, apparent diffusion coefficient of water; T2, mapping of T2 relaxation time; (**B**) Coronal maximum intensity projection of TOF-MRA shows right-sided flow deficit; (**C**) Pictorial representation of the location of the thrombus within the cerebral arterial tree. Coronal slice from 3D T1-weighted images at level of the middle cerebral artery (MCA) origin: the EP-2104R enhanced image (**E**) clearly identifies the thrombus (arrow) that was not visible on the image acquired before EP-2104R injection (**D**). Subfigure **A** is reproduced with permission from *Hilger et al.* [152], whereas subfigures **B**–**E** are reproduced with permission from *Uppal et al.* [45].

Carotid stenosis can significantly affect the blood flow to the brain; therefore hemodynamics are an important parameter in detecting its clinical severity. To investigate this, *Hilger* *et al.* [152] optimized MRI techniques to quantify slow blood flow in thrombotic strokes, a condition often not adequately visualized with standard MR angiography and imaging techniques (Figure 6A). Although the skull is a significant barrier for optical imaging, cranial windows have been used to increase photon penetration depth while using two-photon microscopy to quantify flow in cerebral vasculature [153]. A recent method developed by *Chen et al.* [154] uses time-resolved optical coherence tomography combined with microscopic particle image velocimetry to provide high resolution vascular flow measurements. These measurements vary by as little as 10 μm/s, and quantify the geometry of a single segment of artery at 80 frames per second in real time. Similarly, *Lam et al.* [155] utilized two-photon microscopy and fluorescently labeled emboli to quantify the temporal progression of embolism reabsorption and cerebral flow. Although both of these systems perform well on microvasculature in small animals, the technology’s depth penetration of <1 mm limits analysis of human vasculature. With its better depth penetration, US has been used to reconstruct the 3D lumen geometry of the carotid arteries and thereby detect the degree of stenosis [156]. This technology can also be easily applied to image other parts of the body that may indicate atherosclerotic progression, such as the liver where recent work has shown a correlation between non-alcoholic fatty liver disease and atherosclerosis [157]. This application of ultrasound could be used to study the changes in arterial physiology in the context of stenosis and exemplifies an imaging technique that has clear translational potential.

#### 3.2.3. Molecular Imaging of Carotid Plaques

Advanced imaging modalities have been used to identify thrombus or plaque buildup using targeted contrast agents in rodent models. One common target is fibrin, a clotting protein found abundantly in plaques and clots. For example, *Uppal et al.* [45] combined TOF-MRA and a fibrin-specific contrast agent to simultaneously image fluid flow and plaque formation in the brain (Figure 6B–E). Other groups have targeted fibrin with peptide chains, such as ^111^In-labeled EPeP and FibPep, which serve as tracers for SPECT/CT imaging [81,158,159]. These magnetic nanoclusters serve as negative MR contrast agents, though their tendency to cluster and inability to degrade are barriers to clinical translation.

### 3.3. Cerebrovascular Aneurysms

Cerebral aneurysms are often located within the Circle of Willis, most frequently originating in the anterior cerebral and communicating arteries and to a lesser degree in the lower pressure peripheral arteries, such as the middle cerebral artery [27]. While there are several different types of cerebral aneurysms, including fusiform and dissecting varieties, this review focuses on saccular, or berry, aneurysms as they account for about 90% of all intracranial malformations [160]. Formation of saccular aneurysms is often attributed to an increase in wall shear stress, which often occurs at arterial bifurcations and in more tortuous vessels [161]. As with other aneurysms, systemic hypertension is a major risk factor for development and expansion.

#### 3.3.1. Small Animal Models of Intracranial Aneurysms

Preclinical research over the last decade has used small animal models to study growth, development, and treatment of cerebral aneurysms with direct clinical translational potential [162]. Simple arterial ligation of the common carotid paired with chemical treatments in rodents follows typical cerebral aneurysm progression [163,164,165]. However, invasive manipulation of these vessels is challenging due to the small size of the cerebral vasculature in rodents. Several models employ simple ligation of a common carotid artery in combination with hypertension induction [163,164,165]. Hypertension-induced cerebral aneurysms provide an excellent non-invasive corollary between small animal model and human aneurysm development. Unilateral ligation of the left common carotid artery combined with renal hypertension has been shown to induce saccular aneurysms specifically at the right anterior cerebral artery-olfactory artery bifurcation in rats [164]. The ability to target specific vascular regions is important to accommodate the many variations observed in human anatomical structure. A similar ligation technique can be supplemented with sodium chloride (NaCl) to elevate systolic hypertension and increase the number of lesions created [165]. Additionally, rabbits can be used to test intracranial devices as they mimic some of the geometry and flow patterns seen in human aneurysms [163]. Various sizes of both wide and small-necked saccular aneurysms can be formed through vessel ligation and introduction of intravascular elastase to promote degradation of elastic laminae [163]. Digital subtraction angiograms confirm close homology between experimental models and intracranial aneurysms in humans [166]. While the animal models described have translational potential, a more robust, standardized multicenter study is likely required to optimize further preclinical evaluation of imaging and endovascular device technologies aimed at treating intracranial aneurysms [167].

#### 3.3.2. SAH Imaging

The major clinical concern with cerebral aneurysms is rupture and SAH, a phenomenon that both MRI and CT can diagnose [168]. By combining multiple pulse sequences sensitive to different anatomical regions, such as susceptibility weighted imaging and fluid attenuated inversion recovery, MRI demonstrates notably better detection of SAH than CT alone [168]. Magnetic resonance diffusion weighted imaging in rats is an excellent method to observe the progression of SAH and monitor vasospasms following aneurysm rupture [169], cerebral blood flow, intracranial pressure, and cranial perfusion pressure in real time [170]. All of these parameters are important to consider when studying how the brain undergoes and recovers from SAH.

### 3.4. Cerebral AVMs

While AVMs can be found anywhere in the body, they are of greatest concern within the cerebrovascular circulation due to the risk of intracranial hemorrhage. However, excision of unruptured cerebral AVMs is controversial as it is highly invasive and increases the risk of cerebral hemorrhage and edema [171]. In order to guide clinical decisions, it is important to understand the pathogenesis of AVMs, the effects of abnormal blood flow, and the role hemodynamics play in the progression from cerebrovascular AVMs to intracranial hemorrhage.

#### 3.4.1. Small Animal Models of AVM

Early studies in small animals use surgical anastomoses of the carotid artery to the jugular vein, connecting the intracranial arterial circulation to the extracranial venous circulation, to mimic the changes in cerebral blood flow [30,31,60]. Genetic mouse models of AVMs include those for hereditary hemorrhagic telangiectasia (HHT; Rendu-Osler-Weber syndrome) with mutations in endoglin or activin receptor-like kinase 1 (Alk1) [172,173,174,175,176,177,178] or proteins regulated by Alk1 [62]. Other AVM models have mutations in Notch signaling, responsible for arterial *versus* venous endothelial specification [179,180,181]. Additional genetic mouse models of HHT have emerged using the Cre-lox conditional knockout system. Inducible HHT Cre-lox mouse models have subsequently enabled researchers to postnatally induce Alk1 deletions from mice and simultaneously add a wound or vascular endothelial growth factor (VEGF) stimulus to study their combined effects [174,178]. These genetic models often develop AVMs in many organs, as well as subdermally, facilitating *in vivo* imaging of the dynamic progression of AVM pathogenesis directly below the skin. Extrapolating the findings of subdermal AVMs in genetic models to cerebral AVMs is sometimes necessary as the skull limits the utility of some non-invasive imaging modalities, including standard ultrasound and optical imaging.

#### 3.4.2. Structural and Hemodynamic Imaging of AVM

Structural and hemodynamic imaging has been used to confirm the formation of AVMs and their role in cerebral hypoperfusion in small animal models. Gross morphology, patency, and retrograde flow within an AVM has been quantified by micro-CT angiography [60,62], transcranial Doppler ultrasound [30], fluorescent microspheres [62,174], and fluorescently labeled red blood cells [178] perfused through the vascular beds in small animals. *Morgan et al.* [60] used contrast enhanced micro-CT angiography in surgically-created carotid-jugular AVMs to reveal increased diameter of the anastomosed carotid artery compared to carotids in control rats and a greater blood volume in the Circle of Willis, verifying shunting into the jugular vein. Radionuclide imaging of regional cerebral blood flow has shown that AVMs cause hypoperfusion to the cerebral vasculature [60]. Furthermore, laser Doppler flowmetry measurements showed that this cerebral hypoperfusion remains low for 3 months following surgical AVM creation [31]. Overall, these studies show that AVMs lead to significant changes in hemodynamics, causing retrograde blood flow and chronic cerebral hypoperfusion.

Many studies have also investigated the structural changes in response to occluding the AVM to restore normal perfusion pressure [30,31,60]. *Morgan et al.* [60] used radionuclide imaging to demonstrate that surgical ligation of the carotid-jugular fistula resulted in reactive hyperemia. Previous studies using perfusion of Evans Blue fluorescent dye to label extravascular leakage *ex vivo* have shown that vascular permeability increases after occluding AVMs [30,31]. Together, these studies suggest that the postoperative increase in blood flow and vascular permeability likely reflects the mechanism of brain edema after removal of cerebral AVMs [60].

#### 3.4.3. Optical Imaging and the “Response-to-Injury” Paradigm of AVM Pathogenesis

Although the cause of sporadic AVMs is still unclear, research on small animal models has provided support for a “response-to-injury” paradigm, which speculates that AVM pathogenesis requires a stimulus such as trauma, inflammation, or compression that creates an abnormal angiogenic response [182]. Optical imaging has gained insights into the initiation, progression, remodeling, and hemodynamics within AVMs. Using four-dimensional two-photon imaging to visualize AVMs through cranial windows, *Murphy et al.* [179] discovered that Notch4 normalization causes AVMs to regress. Observations using intravital hyperspectral optical imaging through a skinfold window to measure HbO_2_ saturation showed that the dilation of postcapillary venules (and later vascular remodeling) occurred after AV connections formed [178]. This indicates that an AVM is a risk factor for aneurysm dilation, a finding contrary to previous studies suggesting the opposite relationship [181,183]. Inducible HHT Cre-lox mouse models have been used to trigger Alk1 gene deletions simultaneously with an injury [178] or VEGF stimulus [174] to study the roles of these stimuli in *de novo* AVM formation. Optical imaging in these mouse models demonstrates that an isolated deletion in Alk1 is not sufficient to initiate AVM formation, but rather it requires a simultaneous injury or VEGF stimulus. These results suggest that angiogenesis associated with the wound response is important in AVM pathogenesis in genetically predisposed vessels and may indicate potential therapies, such as anti-VEGF molecules, for halting AVM initiation and progression in predisposed individuals.

## 4. Lower Limb Athero-Thrombosis

Recent imaging developments have provided tools capable of studying biomarkers, hemodynamics, and the natural healing response associated with lower limb PAD. This section will focus on recent US, LDPI, PET, CT, and MRI imaging advancements that have improved our ability to obtain molecular, structural, and blood flow information in the context of lower limb PAD. Critical limb ischemia (CLI), a serious form of lower limb PAD, is caused by severe arterial stenosis that reduces blood flow to the limbs, often resulting in lower-limb discomfort, ulcers, and gangrene [184]. Angiogenesis and arteriogenesis are the two major mechanisms that help supply blood to regions with poor perfusion. Angiogenesis is the process in which new capillaries form from preexisting vessels in ischemic tissue, while arteriogenesis is the expansion of preexisting collateral arteries in response to ischemia [185]. In this section, we discuss the application of imaging technologies to characterize the angiogenic response in ischemic perfusion recovery in small animal models of lower limb PAD.

### 4.1. Small Animal Models for Lower Limb PAD

Small animal models for lower limb PAD that mimics the human progression are needed to understand the impaired processes in ischemic perfusion recovery. *Baltgalvis et al.* [186] used atherosclerosis prone apoE^−/−^ mice to replicate the human lower limb PAD condition, but found no signs of dysfunctional perfusion in apoE^−/−^ mice with confirmed femoral artery plaque. This lack of genetically-induced lower limb PAD models has forced researchers to rely on surgically induced arterial occlusion models that reduce blood flow to the lower extremities. These surgical procedures include a single ligation of either the iliac artery proximal to the internal branch or femoral artery proximal to the superficial epigastric artery [187,188]. A slight variation on this model requires the complete excision of one femoral [187,189,190] or iliac artery [191], or ligation of both femoral and iliac arteries [192]. The single ligation of either the iliac artery or the femoral artery typically results in complete blood flow recovery within a week due to the expansion of already formed collateral arteries [192]. This is supported by *Shireman et al.* [193], who used LDPI to compare the ischemic effect of five different hind-limb surgeries and found that the extent of impaired perfusion recovery increased as the blood flow was progressively interrupted. Both the double ligation and total excision approach have longer recovery times that better mimic human progression, thus requires angiogenesis to play a greater role in perfusion recovery [192].

Interestingly, the current preclinical lower limb PAD models are good approximations of acute limb ischemia, but do not mimic the gradual, chronic decrease in blood flow seen in human disease progression. As a result, compensatory arteriogenesis occurs by different mechanisms, which may be a significant discrepancy affecting the translational potential of discoveries found in these acute models [194]. *Tang et al.* [195] suggested that the sudden blockage of arteries in the acute CLI ligation model causes an abrupt pressure gradient between ischemic and non-ischemic regions, thereby increasing flow and shear stress in collateral arteries and leading to the infiltration of cytokine-producing inflammatory cells that facilitate arteriogenesis. Chronic limb ischemia in humans, on the other hand, leads to the gradual remodeling of muscle tissue fibers, which prevents the shear stress induced inflammation [196]. To better model chronic lower limb PAD progression, an ameroid constrictor is used to gradually occlude the femoral artery in mice [197], rats [195], and rabbits [198]. This model uses a surgically implanted stainless steel ring, filled with hygroscopic casein material that gradually absorbs water and swells, to steadily occlude the femoral artery over a matter of weeks [195,198]. Future CLI studies should use a chronic model, rather than an acute model, to better model human disease progression and understand the body’s recovery and compensation mechanisms in response to CLI.

### 4.2. Factors that Impair Perfusion Recovery

The effects of hyperglycemia, hyperinsulinemia, and hyperlipidemia on perfusion recovery in mice have been studied with LDPI to assess distal limb perfusion, MRI to visualize arterial flow, and micro-CT angiography to evaluate vascular growth. For instance, *Li et al.* [199] and *Hazarika et al.* [200] used LDPI to show that type II diabetic mice, induced with a high fat diet, have decreased perfusion recovery following femoral artery excision. Another group compared arteriogenesis between diabetic, insulin-resistant, and hypercholesterolemic mice, with excised femoral arteries, showing that insulin-resistant and diabetic mice had better perfusion recovery compared to hypercholesterolemic mice [201]. Furthermore, *Tirziu et al.* [59] took a multimodal approach to investigate the impact of femoral artery excision on hypercholesterolemic mice with and without supplementation of an angiogenic growth factor. Their results showed that hypercholesterolemic mice had delayed perfusion recovery, even with angiogenic Ad-PR39 supplementation, due to a decrease in early monocyte and macrophage influx. While the overall degree of recovery was similar, the recovery rate in hypercholesterolemic mice was slower compared to controls. These findings suggested that metabolic impairments decrease perfusion recovery, even when treated with potentially therapeutic angiogenic growth factors.

Other factors such as age, gender, and genetics have shown to impair perfusion recovery in CLI models. *Baltgalvis et al.* [186] demonstrated that older mice have decreased capillary density after induction of hind-limb ischemia, which may be due to impaired endothelial nitric oxide synthase (eNOS)-derived nitric oxide (NO) mechanisms as proposed by *Shimada et al*. [202]. This group used LDPI to track angiogenesis in ischemic Klotho mutant mice, a model for typical aging [202,203], and found that Klotho mutant mice had impaired angiogenesis, as well as decreased capillary density. *Peng et al.* [204] demonstrated that female C57BL/6J mice, induced with unilateral hind-limb ischemia, have decreased perfusion recovery compared to male C57BL/6J mice. While the exact reason for this phenomenon is unknown, the data suggests that the lack of perfusion recovery is a result of impaired capillary formation in female mice. Finally, *Shireman et al.* [193] found that genetic variability between different mouse strains affects tissue necrosis even though the overall perfusion recovery is similar between strains. They also showed that DBA/1J has a significant tissue loss compared to C57Bl/6J and BALB/c mice, which may be useful when studying how genetics influence CLI progression. These studies suggest that metabolic impairment, as well as other genetic variables play a critical role in murine perfusion recovery.

### 4.3. Imaging of Angiogenic Factors in Ischemic Animal Models

**Figure 7:**
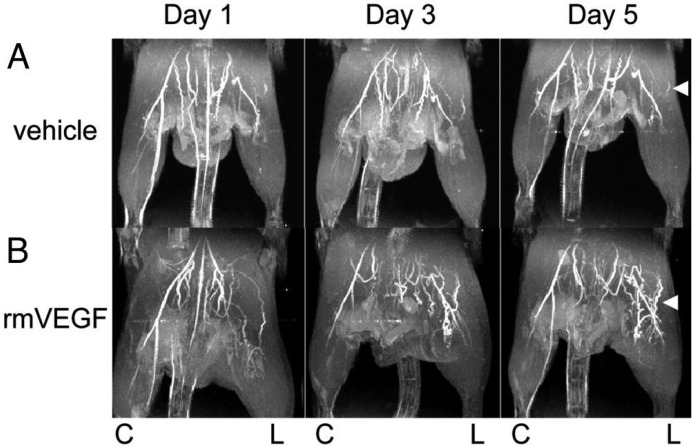
VEGF Improves Collateral Arterial Growth after Hind-Limb Arterial Ligation: TOF-MRA images of ischemic C57BL/6 mice acquired using a small-bore 4.7T magnet. Vehicle (**A**) showed minimal improvement in collateral artery formation, when treated with vehicle control (**B**) showed an increase in collateral arterial formation over 5 days, when treated with rmVEGF. “C” marks the control limb and “L” marks the ligated ischemic limb. Reproduced with permission from *Greve et al.* [206].

*In vivo* imaging of angiogenic ischemic perfusion recovery has proven to be an important strategy to characterize lower limb PAD development. Previous work has shown that downregulation of VEGF can impair artery formation in ischemic tissue [205]. Through LDPI, *Harzarika et al.* [200] showed that hyperglycemia caused poor perfusion recovery in femoral excision models due to impaired VEGF signaling, and *Li et al.* [199] demonstrated that VEGF therapy improves perfusion recovery. Using BOLD MRI, *Greve et al.* [56] also showed that inhibition of VEGF critically hinders angiogenesis in a single femoral ligation ischemia model. Additionally, *Greve et al.* [206] evaluated the effects of recombinant murine VEGF_165_ (rmVEGF) on collateral vessel formation through 3D TOF-MRA (Figure 7). Injection of VEGF_165_ doubled arteriogenesis 5 days after administration, improved angiogenesis, and increased blood flow near the site of injection compared to vehicle controls. Taken together, these studies show that LDPI, BOLD MRI, and TOF-MRA are sensitive techniques to study structural and hemodynamic changes due to angiogenic factors.

While there has been an emphasis on the therapeutic role of VEGF, imaging techniques have also confirmed that other potential factors may impact perfusion recovery. For instance, suppression of eNOS-derived NO expression has been shown to suppress angiogenesis [207]. LDPI studies by *Park et al.* [208] of eNOS^−/−^ and control mice treated with L-NAME, an eNOS inhibitor, showed that eNOS-derived NO is a prerequisite of hemangiocyte recruitment, which has shown to improve perfusion recovery. Others have shown that supplementation with sodium nitrate (NaNO_3_) improved perfusion recovery [209], and calcitonin gene-related peptide can promote angiogenesis [210]. CT and LDPI were used to indicate that deficiencies in certain chemokines can down regulate pro-angiogenic monocytes and thus impair angiogenesis [211]. These findings revealed that imaging modalities can be used to study the effects of several other angiogenic factors, beyond VEGF, on CLI induced perfusion recovery.

One area that must be further explored is the effect of angiogenic factors on perfusion recovery during exercise. Many lower limb PAD patients have adequate perfusion at rest, but only experience inadequate blood flow during movement when oxygen demand is increased. In animal models, the gold standard is the injection of microspheres during exercise [67], which have been used to study the positive impact of exercise training on calf muscle blood flow, arteriogenesis, and angiogenesis after femoral artery ligation [212,213,214]. Other imaging modalities, such as LDPI and CTA, have also been used to study the impact of exercise on perfusion recovery [215]. The benefit of using these imaging modalities is that they allow the study of exercise on specific angiogenic factors and perfusion recovery, thus are an important component of lower limb PAD imaging.

### 4.4. Quantification of Lower Limb PAD Induced Biomarkers

Certain imaging techniques have been improved to track specific angiogenic biomarkers *in vivo*. *Willmann et al.* [79], for instance, developed a Copper-64 labeled VEGF-121 contrast agent to quantify VEGF expression during ischemia, showing that Copper-64-VEGF-121 had increased uptake in murine ischemic hind-limbs. Furthermore, they observed an even greater Copper-64-VEGF-121 uptake after the mice ran on a treadmill [216], which is consistent to the proangiogenic effects of exercise. Behm *et al.* [192] used CEUS to image microbubbles targeted to other angiogenic biomarkers, including activated neutrophils, α_5_-integrins, and vascular cell adhesion molecules (VCAM-1) to assess ischemia-mediated arteriogenesis in ischemic mice with excised iliac arteries. Their results showed that perfusion recovery was accompanied by an increase in α_5_-integrins, a fibronectin receptor that promotes angiogenesis. VCAM-1, a protein that regulates adhesion of monocytes to vascular endothelium, also increased with neutrophil activity. *Leong-Poi et al.* [217] used α_v_- and α_5_β_1_-integrin targeted microbubbles to detect an early angiogenic response to ischemia and to evaluate the effect of intramuscular sustained-release of fibroblast growth factor-2 on blood flow in rats. They found that animals treated with fibroblast growth factor-2 had greater blood flow recovery and produced a greater angiogenic response to ischemia [217]. Together, these studies demonstrated the ability of small animal imaging to quantify and track specific angiogenic and inflammatory responses during ischemia.

## 5. Emerging Technologies and Future Directions

Preclinical PAD research is transforming by continually improving small animal models and imaging techniques. In this section, we highlight two areas driving innovation in imaging development. First, researchers are coupling molecular imaging techniques with novel theranostic particles to study specific PAD disease processes and treatment effects; Second, technical advances in imaging technology are pushing the limits of depth penetration for optical imaging and removing the need for contrast agents in some cases; Additionally, we will discuss the future direction for stem cell therapy in PAD in relation to previous imaging studies.

### 5.1. Theranostic Micro- and Nanoparticles

Current research is focused on exploring the utility of nanoparticles for non-invasive diagnostic imaging and for localized drug delivery. Nanoparticles have been used as contrast agents for MRI [218,219], CT [218], PET/SPECT [220], and optical imaging methods [221,222], and can be functionalized with targeting molecules to localize atherosclerotic plaques. One such method, fluorescence molecular tomography [223], can be used to detect macrophages [224,225] and adhesion molecules within murine plaques [226]. Others have used targeted nanoparticles to image plaques and thrombi using fibrin-binding peptides [81,158,159], smart fluorescent probes that fluoresce upon interaction with proteases [223], and ultrasound-guided echogenic liposomes [227,228]. Additionally, novel dendritic nanoprobes improve PET imaging of ischemic hind-limb mice due to enhanced bioavailability, affinity, and radiostability [229]. Although micro- and nanoparticles have promising applications in PAD, including liposomes loaded with thrombolytics [227] or vasodilators [228] and anti-inflammatory drug loaded nanoparticles [223], issues of production, cost, and toxicity currently limit their translational potential [230]. Similarly, other optically activatable probes have paved the way for intravascular characterization of atherosclerotic plaques, but successful clinical translation necessitates safety and biocompatibility for human usage [231].

### 5.2. Imaging Advancements in Depth Penetration and Resolution

A significant barrier for imaging of PAD progression is developing novel imaging strategies that allow molecular and compositional imaging, while also having a deep penetration depth and high spatial resolution. Some groups have achieved higher depth penetration by combining fluorescence molecular tomography, which uses NIRF probes, with MRI [223] or CT [232], but with limited capacity for resolving tissue heterogeneity [233]. The novel NIRF-II technique [234,235], which uses light with wavelengths between 1.1 and 1.4 μm, has a spatial resolution of approximately 30 µm while also acquiring hemodynamic information from murine femoral artery at rates not detectable with US [236]. Furthermore, multiphoton microscopy is capable of optical sectioning or volumetric imaging of thick tissues. However, vascular imaging is complicated by vessel wall movement due to the cardiac and respiratory cycles [237], meaning multiphoton techniques are mostly used on *ex vivo* tissues or tissues that are easily stabilized. Endoscopic approaches, including a high-resolution two-photon fiber-optic catheter, have helped to gain insight into *in vivo* formation of atherosclerotic lesions, characterization of aneurysmal dilatation, and arterial stenosis [236,238]. A more recent technology, photoacoustic tomography, uses pulsed laser light to generate acoustic signals to obtain high spatial resolution and improved contrast that provides both structural and compositional information with depth penetration of up to 7 cm [239,240]. For arterial disease, photoacoustic tomography is capable of imaging perfusion through microvasculature [241,242,243], identifying lipids [244,245], and differentiating fibrous and lipid components of atherosclerotic plaques [246]. These and other strategies yet to be developed will surely aid in pushing the depth and resolution limits by which current imaging techniques are bound.

### 5.3. Stem Cells as a Treatment of PAD

Advancements in regenerative medicine have shifted the focus of vascular therapies from providing temporary solutions to restoring function at a molecular level [247,248,249]. Mesenchymal stem cells have shown promise to treat both aneurysms and lower limb PAD. The anti-inflammatory and immunosuppressive properties of mesenchymal stem cells on aneurysms, as well as their recruitment in damaged tissues have been well documented in the literature [250,251,252]. Application of mesenchymal stem cells in treating AAAs however requires more thorough investigation. Current treatments for lower limb PAD, including exercise rehabilitation, percutaneous transluminal angioplasty, and surgical revascularization, often prove ineffective as a long-term solution given that 10%–15% of treated patients ultimately undergo amputation [253]. Fortunately, the role of stem cells in neovascularization, arteriogenesis, and angiogenesis has been researched extensively with promising preliminary results [254,255,256]. Bone marrow derived mononuclear cells (BM-MNCs), embryonic stem cells, and mesenchymal stem cells have all been explored as potential treatments for lower limb PAD [254,255,256]. Though each population has its advantages, BM-MNCs have been studied more extensively as they appear to deliver the most promising result in stimulating neovascularization, angiogenesis, and arteriogenesis in ischemic hind-limb murine models for lower limb PAD [233,254,257]. Monitoring blood flow, atherosclerotic plaque deposition, cell viability, and HbO_2_ saturation levels are important when evaluating the efficacy of cell therapy as a potential treatment for lower limb PAD.

**Figure 8:**
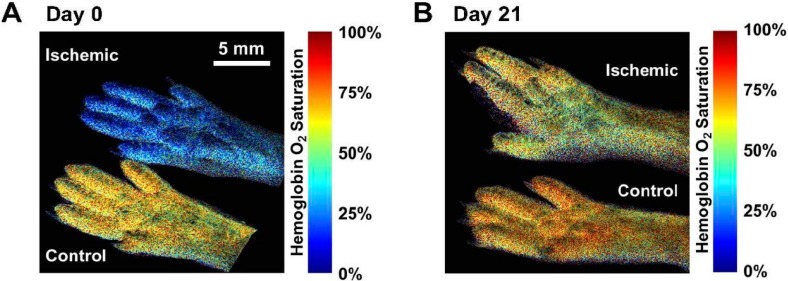
Hyperspectral Ischemic Hind-limb. Ischemic (**top**) and control (**bottom**) hemoglobin oxygen saturation was measured with hyperspectral imaging at (**A**) day 0 and (**B**) day 21 post-femoral artery ligation. Results show a significant improvement in blood flow in the 21-day period. Reproduced with permission from *Poole et al.* [259].

Many of the imaging modalities described previously can be useful when tracking stem cells and determining their effects. Imaging modalities, such as hyperspectral imaging, can provide additional information to assess perfusion recovery (Figure 8). In fact, a combined optical coherence tomography and hyperspectral imaging approach has the potential to monitor the effects of BM-MNCs on peripheral vasculature morphology and blood oxygenation levels, respectively [233]. The results suggested that BM-MNC systemic injection following femoral artery ligation increased both blood oxygenation levels and blood flow. *Van der Bogt et al.* [257] utilized bioluminescence imaging to monitor BM-MNC survival and LDPI to monitor blood flow restoration in the hind limbs following BM-MNC injections. Others have used PET or SPECT imaging to track the distribution of injected radiolabeled stem cells labeled with gallium-68, a radioactive PET agent [258]. While promising, additional imaging research using small animal models will likely be needed to determine if stem cells are a viable strategy for treatment of lower limb PAD.

## 6. Conclusions

Preclinical research in PAD is rapidly evolving with the application and development of novel small animal imaging techniques. In this review, we have examined recent literature describing small animal PAD research and non-invasive *in vivo* imaging of aortic, cerebrovascular, and peripheral vascular PAD small animal models. In particular, this research is improving our understanding of the biological activity, lesion morphology, and hemodynamic alterations in PAD models. Furthermore, monitoring the effects of therapeutics and incidence events, such as vessel rupture or stroke, has been feasible in longitudinal studies. Novel methods for measuring hemodynamic, biomechanical, and molecular changes occurring in PAD are powerful and potentially translatable techniques. Although there are barriers to translation of research findings and novel imaging techniques, the future outlook for PAD research is promising with the growing adoption of small animal imaging supporting these efforts.

## Supplementary Materials

Supplementary materials can be found at http://www.mdpi.com/1422-0067/16/05/11131/s1.

**Supplementary Video S1.** Long-axis B-mode US cine loop (249 frames per second) of an AAA in an AngII apoE^−/−^ mouse. A suprarenal AAA is seen with a point of dissection (blue arrow), thrombotic material (white arrow), and a false lumen (red arrow). Head is towards the left and left renal vein is visible in cross-section on the right.

**Supplementary Video S2.** Short-axis ECG-gated US reconstructed cine loop of an AAA in an AngII apoE^−/−^ mouse. Complex laminar flow is visible in the lumen of the AAA and thrombotic material is evident on the anterior wall of the vessel (white arrow).
